# Ultrasound-guided thoracic paravertebral block combined with dexmedetomidine improves perioperative analgesia and recovery in medical thoracoscopy: a randomized controlled trial

**DOI:** 10.3389/fphar.2025.1684199

**Published:** 2025-11-19

**Authors:** Jia Nie, Wei Chen, Huanhuan Ma, Liang Fang, Zhimin Deng, Yu Zhang, Hai-Ying Wang

**Affiliations:** 1 Department of Anesthesiology, Affiliated Hospital of Zunyi Medical University, Zunyi, Guizhou, China; 2 Department of Anesthesiology, Zunyi Maternal and Child Health Hospital, Zunyi, Guizhou, China; 3 Department of Anesthesiology, Affiliated Xinqiao Hospital of Army Military Medical University, Chongqing, China

**Keywords:** medical thoracoscopy, thoracic paravertebral block, dexmedetomidine, perioperative analgesia, quality of recovery

## Abstract

**Background:**

Medical thoracoscopy (MT) is increasingly performed under local anesthesia with sedation, yet suboptimal analgesia and discomfort remain common and may compromise patient safety, cooperation, and recovery. Ultrasound-guided thoracic paravertebral block (TPVB) provides targeted, long-lasting analgesia, while dexmedetomidine offers cooperative sedation with minimal respiratory depression. Evidence for their combined use in MT is limited. This study evaluated the perioperative efficacy and safety of TPVB plus dexmedetomidine compared with conventional local anesthesia and sedation.

**Methods:**

In this prospective, randomized, controlled single-center trial, 83 patients undergoing elective MT were allocated to receive either TPVB plus intravenous dexmedetomidine sedation (Nerve Block group, n = 41) or standard local infiltration with conventional sedation (Control group, n = 42). Primary endpoints were intra- and postoperative pain scores (Visual Analog Scale [VAS], Behavioral Pain Scale [BPS]) and sedation depth (Bispectral Index [BIS]). Secondary endpoints included Quality of Recovery-15 (QoR-15) score at 24 h, intraoperative physiological stability, drug consumption, adverse events, and other recovery indicators.

**Results:**

Baseline characteristics were comparable between groups. TPVB plus dexmedetomidine provided significantly lower intraoperative VAS and BPS scores across all major procedural stages (all P < 0.05) and reduced postoperative VAS at 1 h, 6 h, and 24 h (all P < 0.05) without rebound pain. BIS values remained within the target range in both groups, with no differences (P > 0.05). The Nerve Block group achieved markedly higher QoR-15 scores at 24 h (median [IQR] 136.0 [124.0–137.5] vs. 127.0 [124.0–129.5]; P < 0.001), exceeding the minimal clinically important difference. Intraoperative hemodynamics, oxygenation, sedative and analgesic drug requirements, and the incidence of hypotension, bradycardia, hypoxemia, or movement were similar between groups, with no major cardiopulmonary or block-related complications. Other recovery outcomes (time to ambulation, gastrointestinal recovery, length of stay, PONV incidence) were comparable.

**Conclusion:**

In patients undergoing MT, ultrasound-guided TPVB combined with dexmedetomidine sedation significantly improved perioperative analgesia and early postoperative quality of recovery without increasing adverse events. This multimodal approach offers a safe, patient-centered anesthetic strategy aligned with enhanced recovery principles and may represent a preferred regimen for MT and other minimally invasive pleural interventions.

**Clinical Trial Registration:**

https://www.chictr.org.cn/, identifier ChiCTR2500098034.

## 1 Introduction

### 1.1 Background and objectives

Medical thoracoscopy (MT) has emerged as a minimally invasive, yet highly effective technique for the diagnosis and management of pleural diseases, including undiagnosed pleural effusions, pleural thickening, and pleural biopsies. Owing to its safety profile and diagnostic accuracy, MT is increasingly utilized in clinical practice worldwide, particularly as an alternative to more invasive surgical procedures (Casal et al., 2009). In the present study, MT refers specifically to a diagnostic or therapeutic procedure confined to the pleural cavity, performed without endotracheal intubation, under local or regional anesthesia combined with light sedation. This approach maintains spontaneous ventilation throughout the procedure and allows patient cooperation, such as adjusting body position when necessary. The term is consistent with the Chinese Clinical Practice Guideline for Medical Thoracoscopy, which differentiates MT from broader non-intubated thoracic surgery (NITS) techniques, including uniportal VATS lung resections.

Traditionally, MT is performed under local anesthesia supplemented with mild sedation in order to maintain spontaneous respiration and facilitate patient cooperation during the procedure (Tousheed et al., 2022). However, perioperative pain and discomfort remain frequently encountered challenges. A substantial proportion of patients continue to experience significant procedural pain, anxiety, and dissatisfaction, which may adversely affect procedural safety, patient cooperation, and overall outcomes (Gravino et al., 2005). These issues underscore the need to optimize anesthetic and analgesic regimens to enhance patient comfort and perioperative experience during MT.

Perioperative pain management and optimization of patient experience remain significant challenges during medical thoracoscopy. MT is most commonly indicated for evaluation of undiagnosed exudative pleural effusions, pleural biopsy, drainage of benign or malignant effusions, and selected therapeutic procedures such as pleurodesis. Contraindications generally include severe hypoxemia, uncontrolled coagulopathy, hemodynamic instability, or the inability of the patient to cooperate with the procedure. A central principle of MT is the maintenance of spontaneous ventilation and adequate oxygenation throughout the intervention, while minimizing patient discomfort and stress (Avasarala et al., 2021; Feray et al., 2023). Inadequate pain control and heightened psychological stress are associated with increased perioperative complications, poorer postoperative outcomes, and reduced overall patient satisfaction (Birnie et al., 2018). Although the combination of local anesthesia and moderate sedation is commonly used, this approach often does not provide sufficiently consistent analgesia; some patients still report substantial pain or discomfort during or after the procedure, revealing the limitations of current protocols (Raft and Richebe, 2019). These shortcomings highlight the need for improved anesthetic strategies that better address both nociceptive and psychological aspects of the perioperative experience during MT.

Advances in multimodal analgesia have led to the increasing use of ultrasound-guided thoracic paravertebral block (TPVB) and dexmedetomidine in thoracic surgery. TPVB enables precise blockade of the ipsilateral somatic and sympathetic nerves, thereby providing effective intraoperative and postoperative analgesia (Yeung et al., 2016). Its application is expanding in thoracic procedures, particularly as part of enhanced recovery after surgery (ERAS) protocols, where it contributes to reduced opioid consumption, improved pain scores, and faster rehabilitation (D’ercole et al., 2018). Dexmedetomidine, a highly selective α2-adrenoceptor agonist, offers a unique combination of sedative, analgesic, anxiolytic, and sympatholytic properties, with minimal risk of respiratory depression (D’ercole et al., 2018). This pharmacological profile is ideally suited for procedures that require patient cooperation or rapid awakening. Previous studies have demonstrated that both TPVB and dexmedetomidine, when used independently, are associated with decreased incidence of adverse events and superior postoperative pain control compared to traditional regimens (Sandeep et al., 2022). These findings suggest the potential value of integrating these modalities into the anesthetic management of patients undergoing medical thoracoscopy.

From both theoretical and clinical perspectives, the combination of thoracic paravertebral block (TPVB) and dexmedetomidine holds promise for optimizing perioperative sedation and analgesia, enhancing recovery, reducing opioid consumption, and improving both patient satisfaction and safety (Lewis et al., 2022). The additive and potentially synergistic effects of regional and systemic multimodal analgesia may provide more comprehensive nociceptive and anxiolytic control during thoracic procedures (Zhao et al., 2022). However, the majority of existing studies have focused on surgical thoracoscopy or major thoracic surgeries such as thoracotomy and video-assisted thoracoscopic surgery (VATS), with only limited evidence addressing the specific context of MT (Hamilton et al., 2022). Systematic evaluations of this multimodal anesthesia strategy in MT are particularly lacking, representing a critical gap in the literature.

Therefore, the aim of the present study is to systematically evaluate the perioperative effects of ultrasound-guided TPVB combined with dexmedetomidine infusion in patients undergoing medical thoracoscopy. By examining this strategy’s impact on intraoperative comfort, perioperative pain control, and postoperative recovery, our study seeks to provide robust, evidence-based recommendations for optimizing anesthetic management in MT and to support broader adoption of enhanced recovery protocols in this patient population.

## 2 Methods

### 2.1 Trial design

This study was designed as a prospective, randomized, controlled, single-center clinical trial. All procedures were conducted in accordance with the ethical principles outlined in the Declaration of Helsinki and applicable local regulations, and reported in accordance with the CONSORT 2010 statement; checklist and flow diagram in Supplementary Material.

### 2.2 Ethical considerations

The study protocol received approval from the Ethics Committee of Affiliated Hospital of Zunyi Medical University, and written informed consent was obtained from all participants prior to enrollment. The trial was registered with the Chinese Clinical Trial Registry (Registration Number: ChiCTR2500098034), and all aspects of patient confidentiality and data integrity were strictly maintained throughout the study.

### 2.3 Participants

#### 2.3.1 Inclusion criteria

Eligible participants were adults aged 18–75 years, of either sex, with an American Society of Anesthesiologists (ASA) physical status I–III, who were scheduled to undergo elective medical thoracoscopy (MT) for diagnostic or therapeutic indications. All patients were required to provide informed consent for study participation.

#### 2.3.2 Exclusion criteria

Exclusion criteria included known allergy or contraindication to local anesthetics, dexmedetomidine, or study-related medications; presence of severe systemic comorbidities (such as decompensated cardiac, hepatic, or renal disease); coagulation disorders; infection at the puncture site; chronic opioid use or substance abuse; significant cognitive impairment or psychiatric illness that would interfere with consent or protocol compliance; refusal to participate in the study; perioperative withdrawal of consent; need for intraoperative blood transfusion; or incomplete or missing perioperative data.

### 2.4 Randomization and grouping

Participants who met the eligibility criteria were randomly assigned to either the control group (receiving standard care with local anesthesia and sedation) or the intervention group (receiving ultrasound-guided thoracic paravertebral block combined with intravenous dexmedetomidine infusion) in a 1:1 ratio. Randomization was performed using a computer-generated random numbers table, and group allocations were concealed in sequentially numbered, sealed, opaque envelopes, which were opened immediately prior to the procedure.

To minimize bias, a single-blind design was implemented such that patients were unaware of their group assignment, while the attending anesthesiologists administering the interventions were informed of the allocation. Data collection and outcome assessments were performed by independent investigators who were blinded to group assignments.

### 2.5 Anesthetic management

#### 2.5.1 Preoperative preparation

All patients fasted for at least 8 h prior to the procedure. Preoperative assessment included a detailed medical history, physical examination, and standard laboratory investigations. Upon arrival in the operating room, standard monitoring was established, including continuous electrocardiography (ECG), noninvasive blood pressure, peripheral oxygen saturation (SpO_2_), and end-tidal carbon dioxide (EtCO_2_) monitoring. Intravenous access was secured, and baseline vital signs were recorded.

### 2.6 Block and sedation techniques

All patients fasted routinely prior to surgery. Upon entering the operating room, a peripheral intravenous line was established, electrocardiography (ECG), pulse oximetry (SpO_2_), and non-invasive mean arterial pressure (MAP) monitoring were initiated. After patients rested supine while breathing oxygen at 5 L/min via a facemask for 3 min, MAP was measured three consecutive times and the average value was taken as the preoperative baseline. Both groups were positioned in the lateral decubitus position (operative side upward) for subsequent procedures, with oxygen supplementation at 5 L/min via facemask.

Control group: Patients received a loading dose of dexmedetomidine (0.2 μg/kg, intravenous bolus) followed by continuous infusion of dexmedetomidine (0.8 μg/kg over 10 min). Upon completion of the loading dose, continuous infusion at 0.2 μg/kg/h was maintained via infusion pump. When the bispectral index (BIS) value was below 70, skin disinfection was performed and local infiltration anesthesia was administered at the surgical site.

Intervention group: Based on the control group protocol, ultrasound-guided thoracic paravertebral block (TPVB) was additionally performed. Patients remained in the lateral decubitus position (operative side upward). When BIS dropped below 70, the skin was disinfected, and under ultrasound guidance, a thoracic paravertebral block was conducted. The puncture site was located according to the intercostal space under ultrasound visualization, and a high-frequency transducer suitable for patient body habitus was used. After probe sterilization, images were obtained to identify the transverse process, pleura, paravertebral space, and thoracic nerve roots. Normal saline (2 mL) was injected to confirm space expansion and pleural displacement. Then, 0.4% ropivacaine (10 mL) was injected into the paravertebral space under ultrasound guidance, and pleural displacement indicated successful block. Local infiltration anesthesia was then performed at the surgical site.

If intraoperative motor agitation (HR < 50/min, respiratory depression) occurred, 0.5 mg midazolam was given intravenously. If inspiratory inhibition was observed, the jaw was lifted and supplemental oxygen was provided via mask. If surgical incision pain occurred (visual analogue scale [VAS] score >4), 5 μg fentanyl was administered intravenously.

For local infiltration at the surgical site, 1% lidocaine (5–10 mL) was injected by the surgeon after skin disinfection; injection time lasted 5 min. The infiltration area included a 3–5 cm zone around the incision site, with injections delivered in three layers (skin, subcutaneous tissue, and intercostal muscles) to ensure complete coverage of the incision and adjacent regions.

At the end of surgery, propofol infusion was discontinued, and patients were transferred to the post anesthesia care unit (PACU). When fully awake and meeting discharge standards, patients were sent to the ward.

Throughout the procedure, continuous monitoring of ECG, blood pressure, SpO_2_, respiratory rate, and, EtCO_2_ was maintained, and all perioperative events and interventions were thoroughly documented.

### 2.7 Outcomes

#### 2.7.1 Baseline and demographic data

Baseline characteristics, including age, sex, body mass index (BMI), American Society of Anesthesiologists (ASA) physical status, major comorbidities (such as hypertension, diabetes mellitus, coronary artery disease), and pre-existing medical conditions were recorded for all enrolled patients.

#### 2.7.2 Primary and secondary outcomes

The primary outcome was the pain score (measured by visual analogue scale, VAS) during lidocaine local anesthesia and during pleural biopsy.

Secondary outcomes included Mean arterial pressure (MAP), heart rate (HR), and peripheral oxygen saturation (SpO_2_) at four predefined time points: T0: upon entering the procedure room; T1: during lidocaine local anesthesia; T3: at skin incision at the start of surgery; T4: during pleural biopsy. These parameters were recorded and compared between groups. Postoperative pain VAS scores at 6 h, 12 h, and 24 h. The VAS was measured using a 10 cm ruler marked on one side with 10 increments, with one end labeled “0” indicating no pain and the other end labeled “10” indicating the most severe, intolerable pain. The Q15 questionnaire score at 6 h postoperatively. Incidence of intraoperative and postoperative adverse events. Total intraoperative sufentanil consumption. Surgeon and patient satisfaction scores.

#### 2.7.3 Data recording timepoints

Data were collected at the following predetermined timepoints: upon arrival in the operating room (baseline), after completion of the block (if applicable), at the initiation of thoracoscopy, intraoperatively at 10-min intervals, at the end of the procedure, and postoperatively at 1 h, 6 h, and 24 h. For adverse event monitoring and recovery parameters, continuous observation was maintained throughout the perioperative period until discharge.

### 2.8 Statistical analysis

All statistical analyses were performed using SPSS (version 29.0) (IBM Corp., Armonk, NY, United States). Sample size estimation was based on the expected difference in mean perioperative pain scores between groups, with a power of 80%, a two-sided alpha level of 0.05. The minimum required sample size was calculated using PASS. Continuous variables were assessed for normality using the Kolmogorov–Smirnov test and are presented as mean ± standard deviation (SD) or median with interquartile range (IQR), as appropriate. Comparisons between groups were conducted using the independent-samples t-test or Mann–Whitney U test for continuous data, and the chi-square test or Fisher’s exact test for categorical data. Repeated measures, such as pain and sedation scores at multiple timepoints, were analyzed using repeated-measures analysis of variance (ANOVA) or the Friedman test, as applicable. All statistical tests were two-tailed, and a P-value less than 0.05 was considered statistically significant.

## 3 Results

### 3.1 Participant flow and baseline characteristics

**FIGURE 1:**
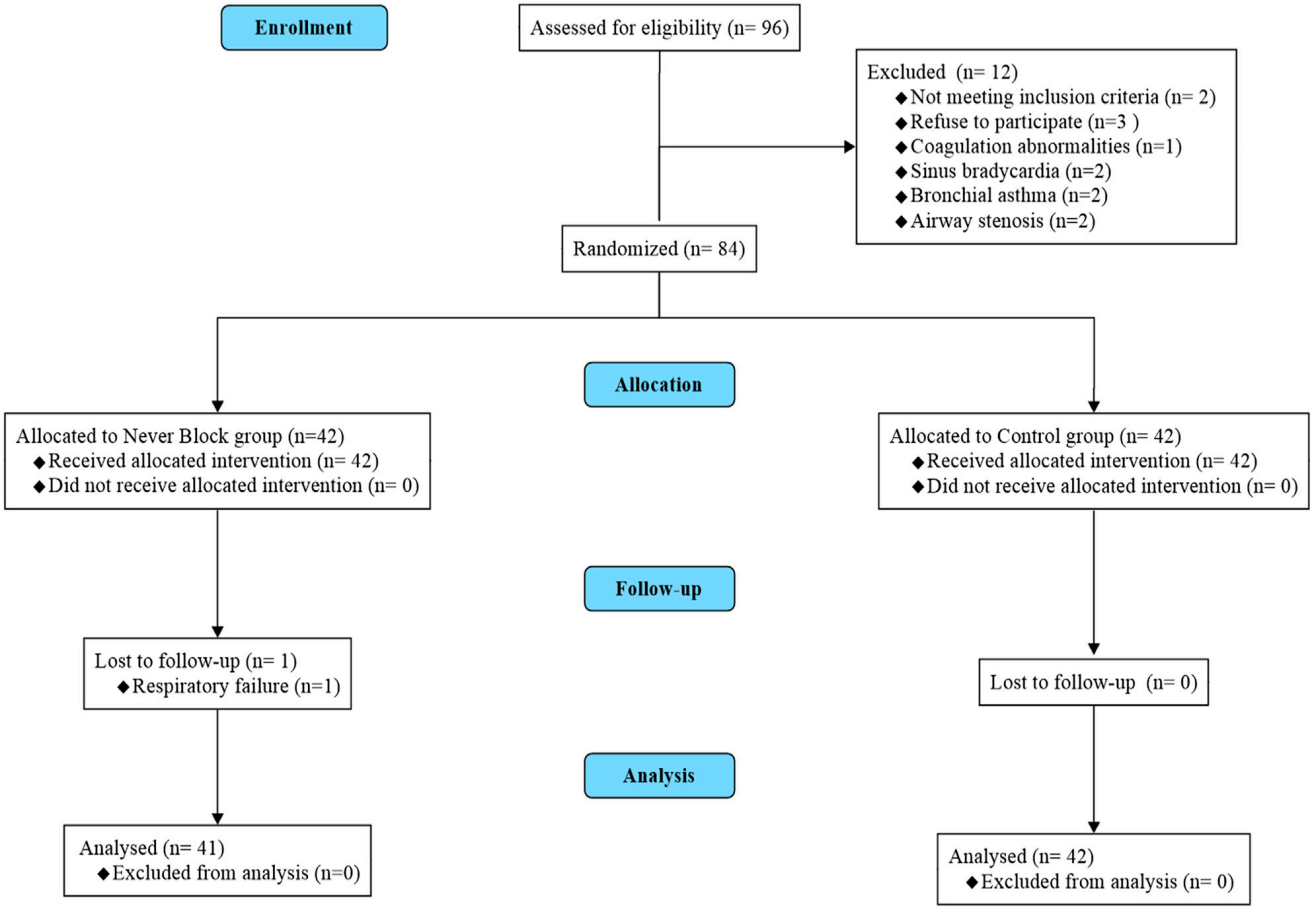
CONSORT flow diagram of patient enrollment, randomization, and analysis. Flow diagram illustrating patient screening, eligibility assessment, randomization, allocation, follow-up, and analysis according to the CONSORT 2010 guidelines. A total of 90 patients scheduled for elective medical thoracoscopy were screened for eligibility. Seven patients were excluded prior to randomization (five did not meet inclusion criteria; two declined to participate). The remaining 83 patients were randomized in a 1:1 ratio to receive either ultrasound-guided thoracic paravertebral block with dexmedetomidine sedation (Nerve Block group, n = 41) or standard local anesthetic infiltration with conventional sedation (Control group, n = 42). All randomized patients completed the allocated intervention, follow-up, and were included in the final analysis; no protocol deviations or losses to follow-up occurred.

A total of 96 patients scheduled for elective medical thoracoscopy were screened for eligibility. After the application of inclusion and exclusion criteria, 83 patients met the requirements and were enrolled. Participants were randomly assigned in a 1:1 ratio to receive either ultrasound-guided thoracic paravertebral block with dexmedetomidine sedation (Nerve Block group, n = 41) or local anesthetic infiltration with conventional sedation (Control group, n = 42). Seven patients were excluded prior to randomization — five due to failure to meet eligibility criteria and two due to withdrawal of consent. All randomized participants completed the intervention and follow-up as per protocol, with no cases of protocol deviation or loss to follow-up (CONSORT flow diagram, Figure 1).

### 3.2 Baseline data

Baseline demographic and clinical characteristics were similar between the two groups, supporting the comparability of the study arms (Table 1). The median age was 49.0 years (IQR 26.0–59.0) in the Nerve Block group and 47.5 years (IQR 36.75–60.0) in the Control group (P = 0.234). The sex distribution did not differ significantly (male/female: 28/13 vs. 12/30, P = 0.756), nor did the median BMI (22.30 [21.05–23.35] vs. 21.50 [18.37–23.82] kg/m^2^, P = 0.144). ASA physical status was predominantly grade II in both cohorts (75.6% vs. 66.7%, P = 0.369).

**TABLE 1:** Preoperative characteristics of the study cohorts.

Baseline characteristics	Nerve block (n = 41)	Control (n = 42)	*P* value
Gender(n), male/female	28/13	12/30	0.756
Age(y)	49.00 [26.00, 59.00]	47.50 [36.75, 60.00]	0.234
BMI	22.30 [21.05, 23.35]	21.50 [18.37, 23.82]	0.144
ASA Grade (n), Ⅰ/Ⅱ	10/31	14/28	0.369
SPO_2_ (%) (pre-procedure)	100.00 [98.00, 100.00]	100.00 [98.25, 100.00]	0.199
SBP (mmHg) (pre-procedure)	122.61 ± 13.31	130.52 ± 4.93	0.014
DBP (mmHg) (pre-procedure)	77.00 [70.00, 84.00]	80.00 [72.50, 82.00]	0.384
MAP (mmHg) (pre-procedure)	91.78 ± 9.54	95.48 ± 8.59	0.067
HR (bpm) (pre-procedure)	84.00 [77.00, 92.00]	80.50 [72.50, 90.00]	0.147
RR (bpm) (pre-procedure)	15.00 [13.50, 17.00]	15.50 [13.00, 17.25]	0.752

BMI, body mass index; SPO_2_: oxygen saturation; SBP, systolic blood pressure; DBP, diastolic blood pressure; MAP, mean arterial pressure; HR, heart rate; RR, respiratory rate.

Pre-procedural vital signs, including SpO_2_, DBP, MAP, HR, and RR, showed no statistically significant differences between groups. The Control group demonstrated a slightly higher mean baseline SBP (130.52 ± 4.93 mmHg vs. 122.61 ± 13.31 mmHg, P = 0.014), but all baseline hemodynamic variables were within clinically normal limits, and no patient required pre-procedural intervention.

### 3.3 Outcomes and estimation: intraoperative hemodynamics and procedural characteristics

**FIGURE 2:**
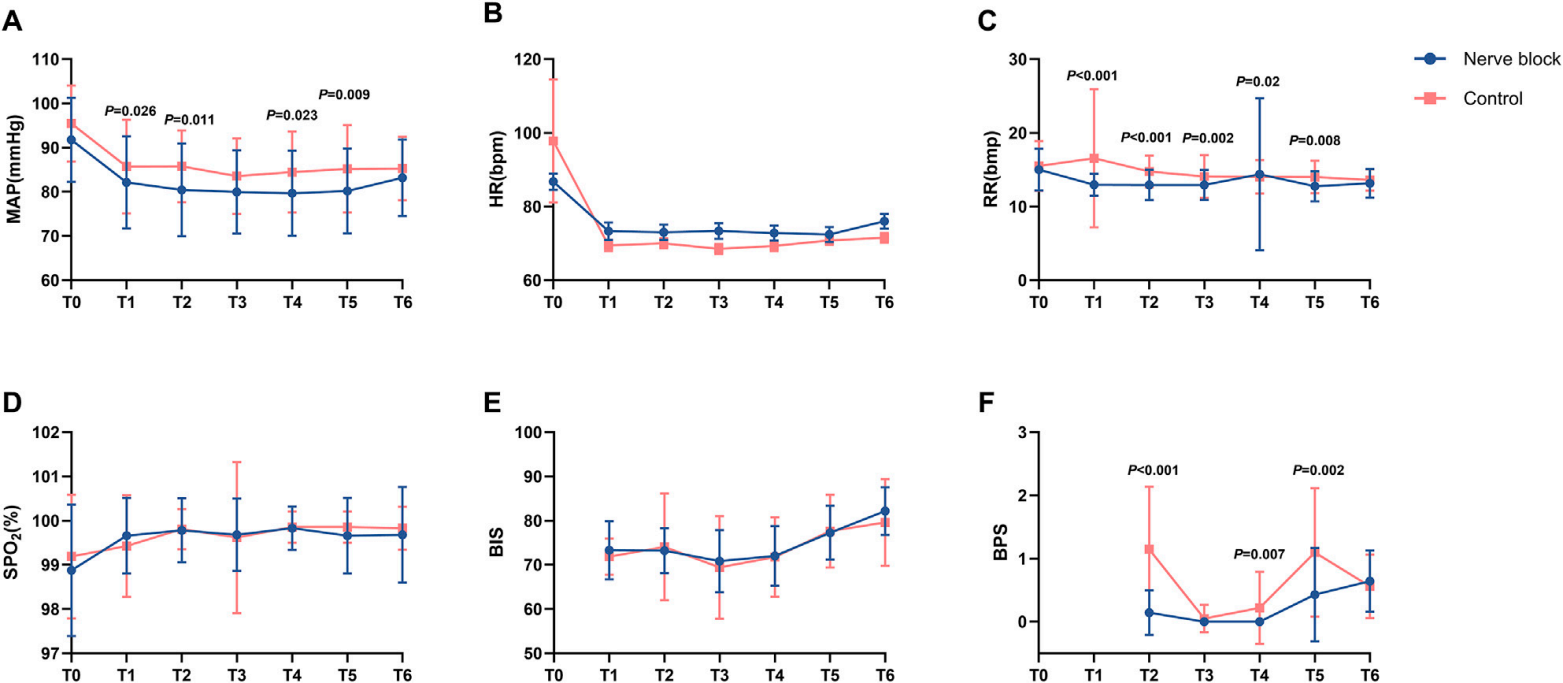
Intraoperative physiological, sedation, and analgesia parameters at predefined procedural time points. Line graphs **(A–F)** showing intraoperative changes in physiological and analgesia-related parameters for the Nerve Block group (n = 41, blue circles) and Control group (n = 42, red squares) at seven standardized time points: T0 = baseline (before induction or block placement); T1 = 10 min after dexmedetomidine loading; T2 = during local anesthetic infiltration; T3 = skin incision; T4 = blunt dissection; T5 = pleural biopsy; T6 = end of procedure. Panel **(A)** Mean arterial pressure (MAP, mmHg). Panel **(B)** Heart rate (HR, beats·min^-1^). Panel **(C)** Respiratory rate (RR, breaths·min^-1^). Panel **(D)** Peripheral oxygen saturation (SpO_2_, %). Panel **(E)** Bispectral index (BIS). Panel **(F)** Behavioral Pain Scale (BPS) score. Values are presented as mean ± SD (MAP, HR, RR, BIS, BPS) or median [IQR] where appropriate. P values indicate statistically significant between-group differences at corresponding time points (see Table 2 for full statistical results). Across all parameters, both groups generally maintained stable intraoperative physiological status and oxygenation, with the Nerve Block group demonstrating significantly lower BPS scores at multiple procedural milestones.

Intraoperative cardiovascular and respiratory parameters — including heart rate (HR), mean arterial pressure (MAP), peripheral oxygen saturation (SpO_2_), and respiratory rate (RR) — were prospectively recorded at seven predefined time points: before induction or block placement (T0), 10 min after dexmedetomidine loading (T1), during local infiltration (T2), at skin incision (T3), during blunt dissection of adhesions (T4), during pleural biopsy (T5), and at procedure completion (T6) (Figure 2).

**TABLE 2:** Intraoperative variables between groups.

Intraoperative monitoring	Nerve block (n = 41)	Control (n = 42)	*P* value
SPO_2_ (%) (10 min post-dexmedetomidine)	100.00 [100.00, 100.00]	100.00 [100.00,100.00]	0.511
SBP (mmHg) (10 min post-dexmedetomidine)	109.00 [100.00,115.00]	118.00 [111.25,127.75]	<0.001
DBP (mmHg) (10 min post-dexmedetomidine)	67.00 [63.00, 77.00]	69.50 [65.00, 75.75]	0.416
MAP (mmHg) (10 min post-dexmedetomidine)	80.00 [76.00, 87.00]	87.00 [80.25, 92.00]	0.026
HR (bpm) (10 min post-dexmedetomidine)	70.00 [62.00, 79.00]	67.00 [64.00, 76.75]	0.455
RR (bpm) (10 min post-dexmedetomidine)	12.00 [12.00, 14.00]	15.00 [14.00, 17.00]	<0.001
BIS (10 min post-dexmedetomidine)	72.00 [69.00, 75.00]	72.00 [69.00, 74.00]	0.633
SPO_2_ (%) (during local anesthesia)	100.00 [100.00, 100.00]	100.00 [100.00, 100.00]	0.617
SBP (mmHg) (during local anesthesia)	105.00 [96.00, 115.00]	118.00 [109.25, 126.25]	<0.001
DBP (mmHg) (during local anesthesia)	68.00 [60.00, 74.00]	70.50 [65.00, 74.00]	0.43
MAP (mmHg) (during local anesthesia)	80.44 ± 10.48	85.79 ± 8.15	0.011
HR (bpm) (during local anesthesia)	69.00 [63.00, 81.00]	69.00 [62.00, 75.50]	0.444
RR (bpm) (during local anesthesia)	12.00 [11.00, 14.00]	15.00 [14.00, 16.00]	<0.001
BPS (during local anesthesia)	0.00 [0.00, 0.00]	1.00 [0.00, 1.75]	<0.001
BIS (during local anesthesia)	75.00 [70.00, 75.00]	74.50 [72.00, 76.00]	0.414
SPO_2_(%) (skin incision)	100.00 [100.00, 100.00]	100.00 [100.00, 100.00]	0.66
SBP (skin incision)	105.80 ± 11.72	115.71 ± 13.16	<0.001
DBP (mmHg) (skin incision)	67.02 ± 9.47	67.59 ± 7.45	0.761
MAP (mmHg) (skin incision)	79.98 ± 9.44	83.57 ± 8.56	0.073
HR (bpm) (skin incision)	69.00 [64.00, 82.00]	68.00 [61.00, 74.00]	0.155
RR (bpm) (skin incision)	12.00 [12.00, 14.00]	14.00 [13.25, 16.00]	0.002
BPS (skin incision)	0.00 [0.00, 0.00]	0.00 [0.00, 0.00]	0.16
BIS (skin incision)	73.00 [70.00, 75.00]	73.50 [70.00, 75.75]	0.909
SPO_2_ (%) (blunt dissection)	100.00 [100.00, 100.00]	100.00 [100.00, 100.00]	0.853
SBP (mmHg) (blunt dissection)	105.80 ± 11.75	116.88 ± 13.08	<0.001
DBP (mmHg) (blunt dissection)	66.76 ± 9.80	68.29 ± 7.99	0.438
MAP (mmHg) (blunt dissection)	79.68 ± 9.65	84.48 ± 9.18	0.023
HR (bpm) (blunt dissection)	69.00 [64.00, 81.00]	70.00 [61.00, 76.75]	0.425
RR (bpm) (blunt dissection)	12.00 [12.00, 14.00]	14.00 [12.25, 16.00]	0.02
BPS (blunt dissection)	0.00 [0.00, 0.00]	0.00 [0.00, 0.00]	0.007
BIS (blunt dissection)	72.00 [70.00, 76.00]	74.00 [70.50, 76.00]	0.605
SPO_2_ (%) (during biopsy)	100.00 [100.00, 100.00]	100.00 [100.00, 100.00]	0.45
SBP (mmHg) (during biopsy)	106.00 [98.00, 114.00]	117.00 [108.25, 127.00]	<0.001
DBP (mmHg) (during biopsy)	68.00 [62.00, 74.00]	70.00 [65.00, 74.00]	0.103
MAP (mmHg) (during biopsy)	79.00 [73.00, 89.00]	87.00 [81.25, 90.00]	0.009
HR (bpm) (during biopsy)	69.00 [64.00, 81.00]	72.00 [64.00, 76.00]	1.000
RR (bpm) (during biopsy)	12.00 [11.00, 14.00]	14.00 [12.00, 16.00]	0.008
BPS (during biopsy)	0.00 [0.00, 1.00]	1.00 [0.00, 2.00]	0.002
BIS (during biopsy)	77.00 [75.00, 80.00]	78.50 [72.00, 84.25]	0.746
S_P_O_2_ (%) (procedure end)	100.00 [100.00, 100.00]	100.00 [100.00, 100.00]	0.839
SBP (mmHg) (procedure end)	109.40 ± 15.78	113.83 ± 15.92	0.207
DBP (mmHg) (procedure end)	69.80 ± 8.12	69.90 ± 6.29	0.950
MAP (mmHg) (procedure end)	83.17 ± 8.65	85.29 ± 7.17	0.228
HR (bpm) (procedure end)	73.00 [66.00, 84.00]	69.00 [65.00, 79.00]	0.164
RR (bpm) (procedure end)	13.00 [12.00, 14.00]	14.00 [12.00, 15.00]	0.115
BPS (procedure end)	1.00 [0.00, 1.00]	1.00 [0.00, 1.00]	0.305
BIS (procedure end)	82.18 ± 5.39	79.62 ± 9.79	0.146

SPO_2_, oxygen saturation; SBP, systolic blood pressure; DBP, diastolic blood pressure; MAP, mean arterial pressure; HR, heart rate; RR, respiratory rate; BPS, behavioral pain scale; BIS, bispectral index.

Across all measured intervals, both groups maintained stable hemodynamic and respiratory profiles without clinically relevant fluctuations. There were no statistically significant between-group differences in mean HR, MAP, SpO_2_, or RR at any time point (all P > 0.05; Table 2; Figure 2). For example, median SpO_2_ at T1 remained at 100.0% [IQR 100.0–100.0] in both groups, reflecting consistently optimal oxygenation and effective maintenance of spontaneous ventilation throughout the procedure.

In terms of perioperative drug administration, total intraoperative doses of local anesthetic, sedatives, and supplemental analgesics were comparable between the Nerve Block and Control groups (all P > 0.05; Table 2). The adherence to standardized anesthetic and monitoring protocols across cases ensured a uniform procedural environment, minimizing the potential for bias due to intraoperative management variability.

### 3.4 Adverse events and safety outcomes

Intraoperative and postoperative adverse events were prospectively monitored and documented for all participants (Table 2). Monitored safety outcomes included patient movement, hypotension (systolic blood pressure <90 mmHg), hypoxemia (SpO_2_ < 90% lasting >1 min), and sinus bradycardia (heart rate <50 bpm), in addition to any evidence of local anesthetic systemic toxicity or block-related complications.

Patient movement during the procedure occurred less frequently in the Nerve Block group compared with the Control group; however, the between-group difference did not reach statistical significance (P > 0.05). The incidences of hypotension and sinus bradycardia were low and comparable between groups, and all episodes were self-limiting or responsive to minimal pharmacological intervention, with no severe or prolonged hemodynamic instability observed in either cohort.

Transient hypoxemia was noted sporadically in both groups, was corrected promptly with oxygen supplementation and airway repositioning, and did not result in procedure interruption or residual sequelae. Importantly, no cases of local anesthetic systemic toxicity, pneumothorax related to the block, or major adverse cardiopulmonary events were recorded.

The addition of ultrasound-guided thoracic paravertebral block with dexmedetomidine did not increase the incidence of intraoperative or postoperative adverse events compared with standard local anesthesia with sedation, confirming the favorable safety profile of this multimodal approach in patients undergoing medical thoracoscopy.

### 3.5 Perioperative pain and sedation profiles

**FIGURE 3:**
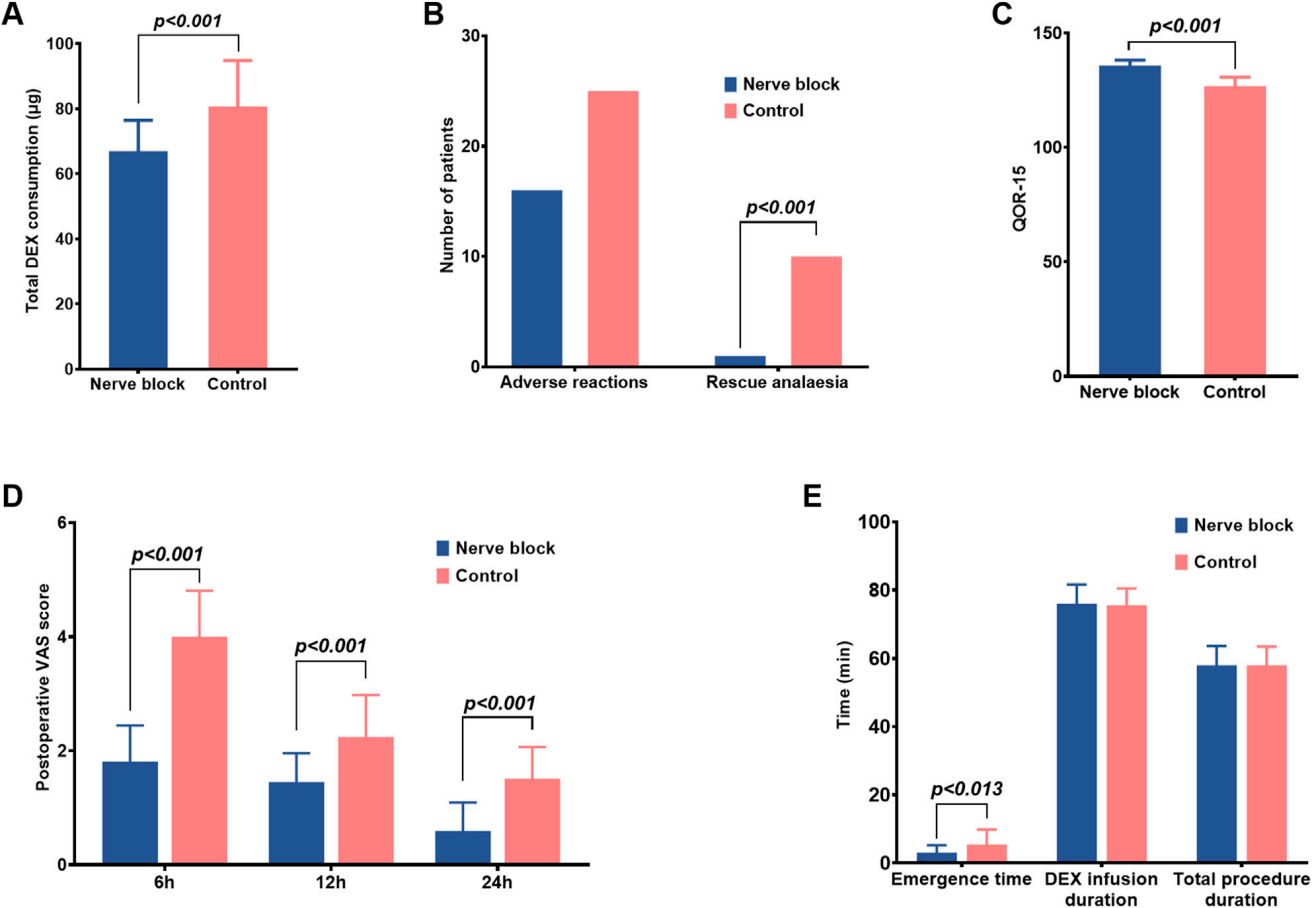
Postoperative recovery, analgesia, and sedation-related outcomes in the nerve block and control groups. **(A–E)** Bar graphs comparing additional recovery-related endpoints between the Nerve Block group (n = 41, blue bars) and the Control group (n = 42, red bars). Data are shown as mean ± SD for continuous variables and as counts for categorical variables. Panel **(A)** Total intraoperative dexmedetomidine (DEX) consumption (μg). The Nerve Block group required significantly lower cumulative DEX doses than Controls (P < 0.001). Panel **(B)** Incidence of adverse reactions and requirement for rescue analgesia. The Nerve Block group showed a markedly reduced need for rescue analgesia (P < 0.001), while the incidence of adverse reactions was similar between groups. Panel **(C)** Quality of Recovery-15 (QoR-15) scores at 24 h postoperatively. The Nerve Block group achieved significantly higher scores, indicating improved early postoperative recovery (P < 0.001). Panel **(D)** Postoperative pain intensity assessed by the Visual Analog Scale (VAS) at 6, 12, and 24 h after surgery. Nerve Block patients reported significantly lower VAS scores at all time points (P < 0.001). Panel **(E)** Emergence time, duration of dexmedetomidine infusion, and total procedural duration. Emergence time was significantly shorter in the Nerve Block group (P = 0.013), whereas infusion duration and total procedure time were comparable between groups. P values represent between-group comparisons at each measurement point.

Perioperative pain intensity and sedation depth were evaluated at predefined intraoperative and postoperative time points using the Visual Analog Scale (VAS), Behavioral Pain Scale (BPS), and Bispectral Index (BIS) (Table 3; Figure 3). Assessments were performed at key procedural milestones — including skin incision, blunt dissection, and pleural biopsy — as well as at 1 h, 6 h, and 24 h post-procedure.

**TABLE 3:** Postoperative outcomes analysis.

Efficacy and safety	Nerve block (n = 41)	Control (n = 42)	*P* value
Emergence time (min)	2.00 [2.00, 3.00]	5.00 [2.00, 8.00]	0.013
Adverse reactions (during procedure) (yes/no) (n)	16/25	25/17	0.063
Physician satisfaction (n), Dissatisfied/Neutral/Satisfied	0/0/41	1/17/24	<0.001
VAS (6 h postoperative)	2.00 [1.00, 2.00]	4.00 [3.00, 5.00]	<0.001
VAS (12 h postoperative)	1.00 [1.00, 2.00]	2.00 [2.00, 3.00]	<0.001
VAS (24 h postoperative)	1.00 [0.00, 1.00]	1.00 [1.00, 2.00]	<0.001
QoR-15	136.00 [124.00, 137.50]	127.00 [124.00,129.50]	<0.001
Rescue analgesia (yes/no) (n)	1/40	10/32	0.004
DEX infusion duration (min)	76.00 ± 5.62	75.55 ± 4.93	0.697
Total procedure duration (min)	58.00 [54.00, 60.00]	57.50 [55.00, 63.50]	0.978
Total DEX consumption (μg)	64.48 [60.83, 74.00]	85.05 [70.77, 91.98]	<0.001

VAS, visual analog scale score; QoR-15, 15-item quality of recovery scale; DEX, dexmedetomidine.

Adverse reaction, Any intraoperative occurrence of body movement (pain-induced), hypotension (systolic blood pressure <90 mmHg), hypoxemia (SpO_2_ <90% for >1 min), or sinus bradycardia (heart rate <50 bpm) was recorded as an adverse event.

During surgery, patients receiving ultrasound-guided thoracic paravertebral block with dexmedetomidine sedation consistently demonstrated significantly lower pain scores than those in the Control group. Median VAS and BPS values were reduced across all major intraoperative stages (P < 0.05 for each comparison), indicating robust nociceptive blockade and superior procedural comfort (Figure 3). For example, at skin incision, the median [IQR] VAS in the Nerve Block group was markedly lower than in the Control group, with corresponding reductions in BPS scores, reflecting diminished behavioural indicators of pain.

This analgesic advantage persisted beyond the intraoperative period. Postoperative VAS scores at 1 h, 6 h, and 24 h were all significantly lower in the Nerve Block group compared with Controls (all P < 0.05; Table 3), demonstrating a sustained benefit in pain control and reduced early postoperative discomfort. The trajectory of pain scores over time suggested more stable analgesia without rebound hyperalgesia following block resolution.

Sedation depth, measured continuously with BIS monitoring, remained within the target range for conscious/moderate sedation in both groups throughout the procedure. Mean BIS values did not differ significantly between groups at any assessed time point (all P > 0.05; Table 3). No episodes of intraoperative awareness (BIS >80 with patient recall) or excessive sedation (BIS <60 with delayed emergence) were reported.

### 3.6 Early postoperative recovery assessed by QoR-15

Early postoperative recovery quality was evaluated using the validated Quality of Recovery-15 (QoR-15) questionnaire at 24 h after medical thoracoscopy (Table 3). Patients in the Nerve Block group achieved substantially higher total QoR-15 scores compared with the Control group (median [IQR]: 136.0 [124.0–137.5] vs. 127.0 [124.0–129.5], P < 0.001). This difference not only reached statistical significance but also exceeded the established minimal clinically important difference (MCID) of 8 points for the QoR-15 scale, indicating a change that is likely to be perceptible and meaningful to patients.

Higher scores in the Nerve Block group were observed across multiple QoR-15 domains — including physical comfort, emotional state, pain control, and independence in activities — reflecting a more favorable early postoperative functional status and overall wellbeing. These findings suggest that the combination of ultrasound-guided thoracic paravertebral block and dexmedetomidine provides measurable benefits for patient-centered outcomes, aligning closely with the principles of Enhanced Recovery After Surgery (ERAS) by facilitating faster return to baseline physical and psychological function.

### 3.7 Other recovery-related outcomes

Secondary recovery endpoints — including length of hospital stay, time to first ambulation, time to return of gastrointestinal function, and the incidence of postoperative nausea and vomiting (PONV) — were also assessed (Table 3). Median length of stay was similar in both groups, with no statistically significant between-group difference (P > 0.05). Likewise, the time to first ambulation and resumption of gastrointestinal function did not differ meaningfully between groups, indicating that the use of ultrasound-guided thoracic paravertebral block with dexmedetomidine neither accelerated nor delayed these aspects of postoperative recovery under the conditions of this trial. PONV occurred infrequently in both groups, and the incidence was comparable (P > 0.05). All episodes were mild, self-limiting, and did not necessitate rescue antiemetic therapy beyond standard prophylaxis.

## 4 Discussion

In this prospective, randomized controlled trial, we demonstrated that the combination of ultrasound-guided thoracic paravertebral block (TPVB) with intravenous dexmedetomidine sedation provided superior analgesia, more stable intraoperative sedation, and enhanced postoperative quality of recovery compared to conventional local anesthesia with standard sedation for medical thoracoscopy. Patients in the TPVB plus dexmedetomidine group experienced lower intraoperative and postoperative pain scores, improved sedation profiles, and higher QOR-15 recovery scores, without an increased incidence of adverse events such as hemodynamic instability, hypoxemia, or bradycardia. To our knowledge, this is one of the first studies to provide robust evidence for the efficacy and safety of a multimodal regional anesthetic-sedation approach specifically in the setting of medical thoracoscopy. These findings fill an important gap in perioperative pain and comfort management for this patient population and highlight the potential for integrating advanced regional techniques with targeted sedation strategies into routine clinical practice.

Our findings confirm the significant advantages of combining thoracic paravertebral block (TPVB) with dexmedetomidine for analgesia during medical thoracoscopy. This extends the established benefits of TPVB from surgical thoracoscopy populations to the field of medical thoracoscopy. Previous studies and recent meta-analyses demonstrate that TPVB provides effective unilateral analgesia and reduces opioid requirements and analgesia-related side effects compared to systemic analgesics or epidural techniques (Sermonesi et al., 2024; Hutchins et al., 2018).

Dexmedetomidine, as an adjunct to regional anesthesia, is associated with anxiolysis, analgesia, and a favorable safety profile, showing enhanced sedation and reduced respiratory depression compared to other sedatives (Weerink et al., 2017). BIS, BPS, and VAS results in our trial indicated that the TPVB plus dexmedetomidine group maintained deeper yet safe sedation and superior pain control, striking an optimal balance between patient comfort and procedural safety. This is in line with current approaches underscoring that tailored multimodal analgesia and conscious sedation strategies yield the best outcomes in thoracic procedures (Schnabel et al., 2010; Janssen et al., 2019).

The improvement in postoperative Quality of Recovery (QoR-15) scores observed in the TPVB plus dexmedetomidine group underscores the beneficial impact of this multimodal approach on early postoperative health status and patient-centered outcomes. Higher QoR-15 scores reflect enhanced physical comfort, emotional wellbeing, pain control, and independence in daily activities. These results are consistent with contemporary Enhanced Recovery After Surgery (ERAS) protocols, which advocate for regional anesthesia techniques and individualized sedation to minimize opioid-related adverse effects, accelerate functional recovery, and facilitate earlier discharge from the hospital (Myles et al., 2016).

Recent studies have demonstrated that interventions improving QoR-15 scores correlate strongly with higher patient satisfaction, reduced incidence of postoperative complications, and better long-term quality of life (Stark et al., 2013). Notably, the integration of regional anesthesia and dexmedetomidine sedation has shown particular value in thoracic procedures, leading to improved mobility, reduced fatigue, and enhanced postoperative self-care abilities (Moorthy et al., 2023). Our findings further support these observations, highlighting the clinical significance of optimizing multimodal analgesia for better convalescence and enhanced quality of life after medical thoracoscopy (Yang et al., 2020).

In this trial, the incidence of adverse events—including hypotension, bradycardia, hypoxemia, and patient movement—remained low in both study groups, with no significant increase in the group receiving ultrasound-guided TPVB combined with dexmedetomidine. Importantly, no cases of respiratory depression or loss of spontaneous ventilation were observed, underscoring the clear advantage of this regional technique and sedation strategy for maintaining respiratory function and hemodynamic stability during medical thoracoscopy (Moorthy et al., 2023). This aligns with previous reports indicating that TPVB and dexmedetomidine, when used individually, are associated with a favorable safety profile, a reduced risk of opioid-related complications, and reliable preservation of spontaneous breathing (Nishizawa et al., 2017).

Compared with earlier studies employing either TPVB or dexmedetomidine alone for thoracic surgeries such as VATS or pleural procedures, our combination strategy offered synergistic effects in both analgesia and sedation, yielding improvements in perioperative experience and patient-reported outcomes (Holm et al., 2022). Previous randomized trials and meta-analyses have suggested that both TPVB and dexmedetomidine individually confer certain benefits over general anesthesia, including reduced pain scores and fewer side effects (Lee et al., 2016). Additionally, our results indicate that a TPVB-dexmedetomidine composite regimen surpasses traditional TPVB-epidural and dexmedetomidine-propofol strategies in terms of safety, recovery quality, and respiratory protection—advantages of great relevance for patients with compromised pulmonary reserve or higher procedural risk. Notably, current guidelines in anesthesia and thoracic surgery increasingly recognize the role of regional anesthesia and multimodal sedation, yet lack disease- or procedure-specific recommendations for medical thoracoscopy; our study provides much-needed data to inform guideline development and optimize standard practice protocols (Sandeep et al., 2022).

Our findings support the wider adoption of ultrasound-guided TPVB in combination with dexmedetomidine for medical thoracoscopy and potentially for other minimally invasive pleural procedures, especially when spontaneous ventilation and a rapid functional recovery are desired (Moorthy et al., 2023). For anesthesiologists and thoracic physicians, this approach offers a clinically practical means to optimize perioperative comfort, minimize adverse events, and improve overall patient satisfaction and safety.

## 5 Limitations

This study has several limitations that should be considered when interpreting the findings. First, the trial was conducted at a single center with a relatively modest sample size, which may limit the generalizability of the results. Second, the patient population represented a specific spectrum of pleural diseases as accepted at our institution, and therefore may not fully reflect the broader population undergoing medical thoracoscopy. Third, the duration of follow-up focused on short-term postoperative outcomes, without assessment of persistent pain, late complications, or long-term quality of recovery.

## 6 Conclusion

In summary, this prospective randomized controlled trial demonstrates that ultrasound-guided thoracic paravertebral block combined with dexmedetomidine sedation provides superior perioperative analgesia, enhanced comfort, and improved short-term recovery quality for patients undergoing medical thoracoscopy, without increasing the incidence of adverse events. These findings support the value of integrating multimodal regional anesthesia and targeted sedation into anesthetic management protocols for MT, offering a promising strategy to optimize patient-centered care and perioperative outcomes. Our results provide important evidence for the adoption of this approach in routine clinical practice and lay the groundwork for further research to refine and extend comfort-focused, evidence-based perioperative strategies in thoracic procedures.

## Data Availability

The original contributions presented in the study are included in the article/supplementary material, further inquiries can be directed to the corresponding author.
